# CRISPR, a Crossroads in Genetic Intervention: Pitting the Right to Health against the Right to Disability

**DOI:** 10.3390/laws5010005

**Published:** 2016-02-18

**Authors:** Shawna Benston

**Affiliations:** Center for Research on Ethical, Legal and Social Implications of Psychiatric, Neurologic & Behavioral Genetics, Columbia University, 1051 Riverside Drive, New York, NY 10032, USA

**Keywords:** CRISPR, CRISPR/Cas9, gene-editing technology, genome-editing technology, genetics, disability, disability rights, law, ADA

## Abstract

Reproductive genetic technologies (RGTs), including gene-editing technology, are being discovered and refined at an exponential pace. One gene-editing innovation that demands our swift attention is CRISPR/Cas9, a system of clustered regularly interspaced short palindromic repeats and a protein called Cas9. As CRISPR and other RGTs continue being developed, we must remain vigilant concerning the potential implications of genetic-engineering technology on our interpersonal and legal relationships. In the face of increasingly numerous and refined RGTs, we must maintain the rights of everyone: potential parents, prospective children, and individuals (both living and prospective) with disabilities. For those who wish to become parents, how should procreation be regulated in light of developing RGTs, especially gene-editing technology? What duties do parents owe their children, and when does such a duty attach? What role should RGTs play in parents’ fulfillment of their duties to their children? This article will contextualize the right to health and what I will term the “right to disability” in the CRISPR/Cas9 landscape. The article will then explore these rights in reference to the “subjunctive-threshold” interpretation of harm. Finally, I will argue that RGTs must be thoughtfully regulated, with such regulations taking into account the opinions of geneticists, bioethicists, and lay people concerning both the right to health and the right to disability.

## 1. Introduction

The 1997 movie *Gattaca* [1] explored the then-science-fiction prospect of a eugenics program that precipitates a society composed of “valids” and “in-valids”. While genetic discrimination is illegal in that imagined society, valids are seen to qualify for jobs over in-valids. Protagonist Vincent Freeman must navigate this world as an in-valid, having been born without genetic engineering; his parents regret their decision to forgo genetic intervention and use it to engineer Vincent’s younger brother, Anton. The film explores the connection between our genes and our identities, as well as both the promises and pitfalls of genetic redesign.

*Gattaca*’s premise is rapidly becoming our reality. With the emergence and refinement of reproductive genetic technologies (RGTs), especially gene-editing technologies like CRISPR/Cas9, potential parents must decide whether—and if so, how—to utilize the technologies available to them, and genetics scientists and legislatures must determine how best to regulate the technologies.

RGTs, including gene-editing technology, are being discovered and refined at an exponential pace. Inevitably, such technologies “offer[] potential parents unprecedented control over the characteristics of their future children” [2], simultaneously raising an “array of ethical concerns about [their] use” [2]. RGTs have been categorized as either technologies that result in “genetic additions, deletions, or modifications that alter an embryo’s DNA” [3] or processes—including selective abortion to eliminate fetuses with unwanted traits [3] or pre-implantation genetic diagnosis (PGD) [3]—used to select or alter traits without directly manipulating DNA. These categories have been termed “by genetic manipulation” [4] and “by selection”, respectively, by professor I. Glenn Cohen, and “direct” and “indirect”, respectively, by law professor Kirsten Rabe Smolenksy. Gene-editing technologies—which are “by genetic manipulation” or “direct” technologies—are garnering wide-spread consideration as scientists and the public alike grapple with their potential ethical, legal, and social implications for every facet of human, plant, and animal species.

One gene-editing innovation that demands our swift attention is CRISPR/Cas9, which “allows scientists to edit the genome, by removing, replacing, or adding to parts of the DNA sequence” [5]. CRISPR/Cas9 works by using “a pair of ‘molecular scissors’ [the enzyme called Cas9] that cut the two strands of DNA at a precise location, so that bits can be added or removed” [5]. After the Cas9 cuts out a mutation, desired DNA stretches (which are synthesized in labs or even bought from companies that prefabricate to order) can be introduced into the location where the mutation has been cut out. This gene-editing technique would give scientists considerable power to alter the genetic programming of plants, animals, and human beings. As Kate Arkless Gray points out, “[w]e’ve actually borrowed the technique—or at least the principle—from nature. Some bacteria use a similar built-in gene-editing mechanism to protect themselves from harmful viruses—a sort of rudimentary immune system” [5]. Indeed, many of the potential uses for this technology involve the study of and therapy for diseases, including cancer (*i.e*., CAR-T cell therapy (see, e.g., [6])), while other potential uses include non-therapeutic trait selection. CRISPR/Cas9 is “the simplest genome-editing tool to work with because it relies on RNA-DNA base pairing, rather than the engineering of proteins that bind particular DNA sequences” [7].

As our society becomes ever more closely aligned with that in *Gattaca*, we must remain vigilant concerning the potential implications of genetic-engineering technology for our interpersonal and legal relationships. While some people have used available RGTs like PGD and will use CRISPR/Cas9 once it is available, others will continue to procreate without genetic intervention. Furthermore, while some individuals with disabilities might use CRISPR/Cas9 for therapeutic somatic gene editing, others might not. Our society will continue to array itself along a spectrum of physical, cognitive, and psychological ability, but the added options for genetic treatment threaten to increase stigma against those who choose not to use it. In the face of increasingly numerous and refined RGTs, we must maintain the rights of everyone: potential parents, prospective children, and individuals (both living and prospective) with disabilities.

Indeed, our vigilance concerning the rights of individuals with disabilities acquires new pragmatic and philosophical dimensions as the interventional spectrum widens. Although our society has long feared the slippery slope from reproductive and genetic technologies to eugenics, we now fear as well the slippery slope to more widespread disability as an unintended consequence of genetic technology. This fear of iatrogenic disability undergirds many scientists’ and ethicists’ desire to rein in the research and/or application of CRISPR. But while we might imagine that the only CRISPR-related disabilities to be feared are those resulting from unwanted side effects of the technology, in fact gene editing can allow for individuals’ actual choice to engineer disability in themselves or in their offspring. We have seen other RGTs, such as PGD and selective abortion, used in certain cases to facilitate parents’ selection of disability in their offspring, and we note the increasingly researched and debated (see, e.g., [8]) phenomenon of adults’ intentionally disabling themselves surgically or chemically (for a discussion on apotemnophilia, see [9], see also [10]; for a case of intentional self-blinding, see [11]). It is hardly fanciful, then to consider the counter-intuitive uses to which CRISPR/Cas9 may be put as a method of trait engineering potentially capable of addressing disability in a precise, efficient, and relatively affordable manner. Our continuing debate about whether—and, if so, how—CRISPR should be used and regulated must consider a spectrum of therapeutic approaches running from intentional reduction of disability, to non-interference, to the intentional induction of disability. We must ask, then: what would constitute appropriate use of CRISPR to enhance health choices without implicitly devaluing individuals who have *not* received CRISPR therapy? Should limits be placed on the parental exercise of non-interference? In addition, what limits, if any, should be placed on individuals seeking CRISPR to *engineer* disabilities in themselves or their offspring?

Naturally, a tension arises among rights: the right of prospective children to an “open future”, the right of already living and prospective people to disability accommodations, and the constitutional right to privacy in marriage and family. Perhaps the most difficult tension is that between the right to health and what this article will term the “right to disability”.

The notion of a *right to disability* is a philosophical one, yet it is a direct implication of the Americans with Disabilities Act (ADA) [12], which delineates and protects the right of individuals with disabilities to reasonable accommodations, whatever the etiology of the disability. That is to say, the law’s protections encompass not only individuals with unavoidable disability but also individuals who abstain from medical cures, individuals whose parents decline medical interventions on their behalf, and even individuals whose disability was *engineered* by themselves or their parents. To be sure, parental choices made in favor of disability can be challenged legally as constituting *harm* to the child; but particularly in a society where identities and communities are actively reconstituting themselves around what were previously understood as “disabilities”, the legal and ethical understanding of “harm” becomes increasingly tentative (see generally [13], see generally [14]).

The fluidity of the term “disability” is manifest when, for example, individuals with the so-called impairment of deafness reject the notion of being disabled, instead considering deafness a linguistic trait [15]. In essence, disability in one context can be functionality in another. Most broadly, this contextual fluidity is emphasized by the social model of disability, which understands “disability” to indicate a mental or physical impairment that inhibits one’s activities *only if* accommodations are *not* made [15]. That model is enshrined in the United Nations’ Convention on the Rights of Persons with Disabilities (UNCRPD), which declares that “disability results from the interaction between persons with impairments and attitudinal and environmental barriers that hinders their full and effective participation in society on an equal basis with others” [16]. In view of this pronouncement, we might consider speaking of the ADA’s protecting a “right to impairment” rather than a “right to disability”. However, it is striking that neither the UNCRPD nor the ADA offers separate nomenclature for “persons with disabilities” who *have* received appropriate modifications of “attitudinal and environmental barriers”. In other words, the term “disability” seems to apply for the duration of the impairment, whether or not hindrances to inclusion have been eliminated. Do the UNCRPD and ADA inadvertently contradict the social model of disability? I would argue that the stability of their nomenclature actually reflects a recognition that impairment often entails perpetual struggle with attitudinal and environmental barriers. The ideals inscribed into the UNCRPD and ADA are understood as requiring incessant defense; “persons with disabilities” are understood as remaining such in relation to the dominant society. In that light, we *must* denote the ADA’s indifference to the etiology of disability [17] as a “right to disability”—that is, an individual’s right to incur an impairment that will incur perpetual, if inconclusive, efforts by society to remediate the various kinds of barriers associated with the impairment.

Without denying the possible finding of parental liability in any given case, we can view the ADA as creating an environment hospitable to the refusal of medical means for averting disability, and correspondingly an environment hospitable to the use of medical means for inducing disability. I am arguing, then, that the law implies a *right to disability* as an essential component of individual autonomy, a right that extends to the informed consent to use, or not use, RGTs. This article does not advocate a particular set of uses for or restrictions on CRISPR/Cas9 technology, either to “enhance” already healthy embryos and people, or to create disability. Instead, it invites a close look, in light of the ADA and UNCRPD, at implications of CRISPR that need to be addressed at the very time that issues of protection and harm are being hashed out.

One avenue of discussion must concern what goals we, as a society, might have for the destigmatization of disability. While the ADA works to protect civil rights for individuals with disabilities, neither it nor similar legislation in the US (such as the Individuals with Disabilities in Education Act [18]) goes nearly as far as does the UNCRPD in protecting such individuals against social stigma. It seems clear that the US, by means of “more vigorous interpretation and/or action by Congress” [18], can elevate its legal protection for people with disabilities to match the UNCRPD’s. But this move must entail a greater understanding of what the repercussions of stigma reduction or elimination might be. If we are to say—both socially and legally—that disability must be represented as equivalent to health, then we have no basis for telling parents not to choose so-called disabling traits for their offspring. Even if the US does not ratify the UNCRPD but merely views it as the gold standard of legal protection of disability rights and prevention of social stigma, we would still need to anticipate CRISPR’s being used in ways that might initially strike us as unforeseeable and even unpalatable.

Indeed, the *right to health* and the *right to disability* are in effect two sides of the same coin. In our disability-fearing culture it seems easier, at face value, to support the protection of health than the autonomy to maintain or even create disability. But just as the pursuit of health can entail a wide array of approaches, so, too, does the fortification of disability rights. As sociologist Tom Shakespeare elaborates in his book “Disability Rights and Wrongs Revisited”, while the goals of disability rights and medical cures for the so-called disability “problem” are not necessarily mutually exclusive, much nuance is required to determine how best to reconcile these seemingly opposed approaches to helping individuals with disabilities [19]. Disability rights activists and individuals with disabilities harbor diverse points of view, with some fully supporting potential medical cures, including gene therapy; others focusing solely on the removal of social barriers; and still others championing a balance of medical and social interventions. Shakespeare offers the reassurance that “medical cure or therapy [is not] incompatible with social change and civil rights: rather than seeing these as alternative strategies, it is possible to see them as complementary”[19]. He affirms the beneficial coexistence of the equally important rights of health—including access to medical intervention to create or maintain health—and of disability, including reasonable accommodations delineated and protected by the ADA. In effect, I am suggesting that the ADA, while clearly working along the social-intervention axis, also tacitly protects disability itself by ensuring support of the outcomes of medical-intervention choices.

This article will contextualize the complementary yet ostensibly competing rights to health and to disability in the CRISPR/Cas9 landscape by exploring the relevant current debates surrounding this new gene-editing technology. The article will then explore the “subjunctive-threshold” interpretation of harm, which essentially pits these rights against each other as we grapple with determining a “reasonable threshold of harm” above which our offspring should live. Finally, the article will delve more deeply into the meaning and implications of a *right to disability*, and in particular its implications for social stigma (an issue highlighted by the UNCRPD), with consideration for what our society can deem “disabilities” if and when CRISPR is used to eliminate or select for particular traits. I will pose, in closing, a series of questions concerning both the right to health and the right to disability—questions on which the opinions of geneticists, bioethicists, and lay people must be sought in order for CRISPR to be ethically regulated.

## 2. Gene-Editing Technology Debates

The foregoing discussion offers the “*right to disability*” as a lens through which to discern the possibility of what we might call “designer disability”. This consideration takes its place in an already active debate in the scientific and bioethical communities concerning two potential outcomes of CRISPR: over-enhancement of offspring and undesired disabling side effects. Because of the diversity of its applications—as well as its precision, ease, efficiency, and inexpensiveness—CRISPR/Cas9 has aroused considerable ethical concerns among geneticists and bioethicists. This technology could be applied not only to somatic cells as gene therapy (in order to, for example, eradicate a genetic disease such as cystic fibrosis), but also across the germline to alter heritable traits. The latter application could, like the former, be used to eradicate disease; however, it could also be used to create so-called designer babies with preferred physical and potentially even intellectual and emotional traits. Of fundamental concern to many geneticists is how to properly apply this distinction between genome editing in somatic cells and in germ cells [7,[20]. While some scientists, including one of the co-discoverers of CRISPR/Cas9 [21], find this distinction of utmost importance, with vastly heightened concern placed on potential editing of germ cells, others, such as those in the Hinxton Group, emphasize the value of engaging in CRISPR research even in the context of reproductive application because to avoid doing so for fear that such an application is “premature and dangerous will ensure that it remains forever premature and dangerous, for want of better knowledge” [22].

While this technology is not yet regulated in the United States and many of its applications remain a long way from being a reality, Michael Hanlon points out that “scientists are right in saying we should be having this debate now” [23]. Scientists’ explicit invitation to debate is warranted especially in light of calls by industry bodies for a moratorium on even basic research [7,19]. The National Institutes of Health issued a statement explaining that they will not be funding “any use of gene-editing technologies in human embryos” because of “serious and unquantifiable safety issues, ethical issues presented by altering the germline in a way that affects the next generation without their consent, and a current lack of compelling medical applications justifying the use of CRISPR/Cas9 in embryos” [25].

Among the questions under debate are who should be the participants in the debate itself, what constitutes designer babies and eugenics, and how we can address subsequent generations’ lack of consent to germline alteration of their embryos. The latter two concerns, of course—the issue of consent and designer babies—originate in the potential use of CRISPR/Cas9 to alter the germline.

The debate about consent must begin with the recognition that subsequent generations cannot give consent to their genetic inheritance whether passed down to them through natural procreation or through gene-editing technology. The concern about lack of consent is perhaps best understood as originating in fears about the as-yet unpredictable side effects of the technology. If side effects come to light decades––even generations––after the first use of the technology, there may be exponential growth of their prevalence in the population. Thus, concern about side effects is itself subject to a multiplier effect: “gene therapy whose risks are made apparent two, or three generations down the line would be a whole different ball game. A patient can consent to treatment; her great-great grandchildren cannot” [23].

Concern about designer babies presents itself in even more complex and historically rooted questions: Could this technology fall down a slippery slope to eugenics?

The invitation to the lay public to participate in this debate must not be disingenuous: bioethicist J. Benjamin Hurlbut reminds us that since the scientific retreat held at Asilomar, California, in 1975, the role of the public, as perceived by scientists and as played out through scientists’ manipulation, is that of mere dependents [26]. However, the tide may be ready to change: interestingly, while the post-Asimolar “notion [was] that when the public baulks at worrisome research, the appropriate action is to hit ‘pause’ long enough to allow public views to come into closer alignment with scientific judgments”, now we are seeing the scientific community itself—even one of the discoverers of CRISPR/Cas9 [21]—balk at the technological advancements and their implications. Because “an informed deliberation on genetic engineering research and its applications need not depend on comprehensive public understanding of the science behind CRISPR/Cas9 gene editing” [26], it makes sense to reach beyond the scientific community’s concerns and hear what hopes and fears the public has for the technology’s eventual use.

To invite the public’s contribution to the CRISPR/Cas9 debate is implicitly to encourage the drawing of subjective distinctions: first, between states in which life is and is not worth living, and second, between states in which life is satisfactorily worth living and those in which it is optimally worth living. When engaging in discussion of these distinctions, we inevitably ask where—not whether—we should draw a line demarcating disabilities that are worse than either death or no life to begin with. While we may agree with many commentators that no such line should exist, that life is intrinsically valuable and better than no life at all regardless of any physical or mental debilities (see, e.g., [27]), CRISPR invites us to imagine a panoply of designed outcomes, some of which we might personally wish to avoid at any cost. CRISPR thereby prompts us to consider whether we owe a duty to prospective offspring to desist from designing conditions that we deem untenable—in other words, whether we must take on the duty of denying certain kinds of life, or life itself, to a genetically specific individual. This approach stands in contrast to the safeguards placed on assisted dying, which cannot be undertaken in any jurisdiction unless individuals declare that their debilitating conditions render their lives subjectively not worth living (see, e.g., [28]). Our hypothetical duty to the unborn would thus seem antithetical to our duty to the living.

Even setting aside this uneasy preemption of the unborn individual’s self-assessment, we face inevitable tensions between choices that will be made by individual parents and those that will be collectively advocated by the public. Any attempt to use the public’s—or, indeed, the scientific community’s—input to develop general principles constitutes a slippery slope toward imposing certain values on every member of society. Not only would the freedoms of individual dissenters be imperiled but the very process of generating principles would be harmful to all: the drawing of subjective distinctions risks creating a public appetite for hierarchical valuation of other lives; furthermore, this valuation might prime a surge in demand for genetic technology. Thus, the invitation to the public must be extended not only sincerely but with extreme caution. In a sense, we face an infinite regress whereby the desire to consult the public begets the wish to obtain informed consent for such consultation.

Such anxieties notwithstanding, insight into public views at the moment could help scientists and legislators themselves “press the pause button” just long enough to consider how gene-editing technology is likely to be sought therapeutically (*i.e*., to help eradicate genetic disease), how it may be sought to “improve” an otherwise healthy embryo, and whether—and, if so, how—it might be used to engineer disability.

## 3. The Right to Health and an “Open Future”, and the Subjunctive-Threshold Interpretation of Harm

When considering the notion of a right to disability and CRISPR’s potential role in engineering disability, we inevitably encounter a formidable limitation: the subjunctive-threshold interpretation of harm. This theory asserts that “having acted in a certain way (or having refrained from acting in that way) at time *T_1_*, we thereby harm someone only if we cause this person’s life to fall below some specified threshold” [29]. By extension, “a future person can be harmed by us when our choice of actions and policies causes her life to fall below some reasonable threshold of harm, even if this choice is the necessary condition of her coming into existence” [29]. The implication is that “the prospective person has the *right to live above that reasonable threshold*, and that if her existence would place her below that threshold, she should instead be prevented from coming into existence” [30]. Furthermore, “[i]f we assume that future people have general (human) rights vis-à-vis us, our correlative duties set a normative framework for most of our decisions concerning future people” [29].

The subjunctive-threshold interpretation of harm, therefore, involves a rights-duties analysis: the prospective child has the *right* to a life above a predetermined “reasonable threshold” and the potential parents and their doctor owe the prospective child the *duty* to ensure such a life. To say that any life is better than no life at all is to deny prospective children’s right to health, threatening a slippery slope to general parental neglect. Therefore, this article rejects the notion that, from the standpoint of parental duty, any life—however physically or emotionally disordered—is better than no life at all, instead espousing the subjunctive-threshold interpretation of harm. There must be an identifiable “reasonable threshold” for prospective life, with corresponding duties lying in potential parents prior to their children’s conception.

We are therefore faced with pressing questions: how can CRISPR protect prospective children’s rights to health and to disability? How will the introduction of this technology shift the “reasonable threshold of harm”? If we can establish a right to disability, does society harbor corresponding duties beyond the ADA’s (and other relevant laws’) legal protection of individuals with disabilities?

When we talk about rights, we must also examine corresponding duties (see, e.g., [8]). It is not enough to observe that Person A has the right to X; if Person A has the right to X, by dint of that right he must have a relationship with Person B, who owes him a duty to provide him with X. Such a corresponding duty might be either positive or negative: a positive duty would entail the active provision of X, and a negative duty the forbearance of interference with Person A’s acquiring X (see, e.g., [9]). The questions, then, that must arise are: what qualifies as a right, what are such qualified rights’ corresponding duties, and in whom do such duties lie?

If we are to understand that human dignity, equality, freedom, and justice are rights, the corresponding duty might lie in a common authority—a state—to enforce those rights. When a right has been formalized as a law, the result is the state’s positive duty to enforce—to guarantee—that right. The intertwining of personal (*in personam*) rights and their codification into law has resulted in the formula, “[t]he more freedom, the more rights; the more rights, the more laws and state; the more state, the less freedom; the less freedom, the more need of rights *etc.*” [33].

When a putative right has not (yet) been formalized as a law, we might look to the Constitution to determine whether the right extends from a precept therein. Indeed, constitutions, as well as the Charter of Fundamental Rights of the European Union, have deliberately kept certain rights, such as human dignity, free from concrete definition (see, e.g., [10]). With “human dignity” understood both as “inviolable” [34] and as the “normative basis for all basic rights” [33], we find in this right the “ultimate foundation of the claims of right we are entitled to have as individuals, and whose protection must be unconditionally guaranteed in an interpersonal way, and in a collective, political, social and cultural way” [33]. In considering the rights of parents, progeny, and personal seekers of genetic intervention, we must ask: What constitutes “human dignity”, or an extension thereof, such that the given intervention will be protected by law? In other words, what can be classified as a right guaranteed by a positive duty lying in the state?

One such right is the right to privacy in marriage and, by extension, family—a right “older than the Bill of Rights” [35]. The pivotal 1965 case of *Griswold v. Connecticut* found the Court investigating the question of whether a person could use “any drug, medicinal article or instrument for the purpose of preventing contraception” [35]. The Court found that a law prohibiting a person from using contraception was invalid because it violated the “right to marital privacy” [35]. While the U.S. Constitution does not specifically mention a privacy right, Justice Douglas wrote for the majority that “specific guarantees in the Bill of Rights have penumbras, formed by emanations from those guarantees that help give them life and substance” [35].

Crucially, Justice Douglas cited the First, Third, Fourth, Fifth, and Ninth Amendments to explain that *Griswold*

concerns a relationship lying within the zone of privacy created by several fundamental constitutional guarantees. And it concerns a law which, in forbidding the use of contraceptives, rather than regulating their manufacture or sale, seeks to achieve its goals by means having a maximum destructive impact upon that relationship. Such a law cannot stand in light of the familiar principle, so often applied by this Court, that a governmental purpose to control or prevent activities constitutionally subject to state regulation may not be achieved by means which sweep unnecessarily broadly and thereby invade the area of protected freedoms [35].

In other words, a legal prohibition—as distinct from a regulation—of a medical practice that is conducted within an “area of protected freedoms” is unconstitutional.

The impact of *Griswold* proved wide-ranging. Hard on the heels of *Griswold* were the pivotal cases of *Eisenstadt v. Baird* and *Roe v. Wade*, the former extending the right to use contraception to unmarried couples [36] and the latter legalizing abortion for any reason through the first trimester (with possible restrictions in the second and third trimesters) [37].

Thus, we can see a clear line of Supreme Court cases protecting and regulating the right to contraception and abortion—the right *not* to be parents. By the same token, *Griswold* and its ensuing line of cases also establish an extension of the privacy right into the decision to procreate, at least in the absence of assisted reproductive technologies (ARTs) or RGTs [38]. Ongoing debate surrounding a putative right to procreate using ARTs or RGTs concerns both the ethics of such technologies and the potential regulation of them. There is legal precedent supporting both sides of the aisle—either a finding of the technologies’ inclusion in the right to procreate as an extension of procreative liberty (see, e.g., [39–42]) or a finding of their exclusion based on the need for the integrity of the medical profession and/or the need to respect unborn life (see, e.g., [43]). Despite the unpredictability of both lower courts and the Supreme Court [42], some commentators, such as John Robertson, have proven particularly persuasive in their championing of such technologies as extensions of procreative liberty. While acknowledging the fear of gene-editing’s slippery slope to eugenics, on the one hand, and to “perversely” [42] engineered disability, on the other, Robertson nevertheless finds “the case for genetic engineering to produce children…plausible and even compelling” [42].

Likewise, John Harris, in supporting what Ronald Dworkin termed the right to “procreative autonomy” [43], asserts that “the sorts of freedoms that freedom of religion guarantees, freedom to choose one’s own way of life and live according to one’s deeply held beliefs, are also at the heart of procreative choices” [44]. Harris clearly states that “[i]n so far as decisions to reproduce in particular ways or even using particular technologies constitute decisions concerning central issues of value, then arguably the freedom to make them is guaranteed by the constitution (written or not) of any democratic society, unless the state has a compelling reason for denying them that control” [44].

Other commentators, such as Julian Savulescu, have found an ethical impetus for the use of ARTs and RGTs to “select the best children” [45]. Savulescu’s term “procreative beneficence” has at its core a rights-duties bent: While not quite overlapping with the subjunctive-threshold interpretation of harm, which assumes a universal, or at least a societal, threshold of harm below which we should not let our children live, Savulescu’s procreative beneficence urges parents to “select the child, of the possible children they could have, who is expected to have the best life, or at least as good a life as the others, based on the relevant, available information” [45]. Furthermore, Savulescu insists that we collectively have a “moral obligation to test for genetic contribution to non-disease states such as intelligence and to use this information in reproductive decision-making” [45].

Though it remains to be seen how the Constitution will be interpreted by courts in the gene-editing-technology context, Harris’ legal reasoning will likely help provide an analytical basis for regarding technological intervention as an element of procreative choice, with Savulescu’s moral reasoning paving the way for an intensive exploration of the rights and corresponding duties of the parent-child relationship. Indeed, throughout the debate surrounding ARTs and RGTs, especially in light of emerging gene-editing technology, we must continue to examine what duties parents owe their children, when such duties attach, and what role RGTs *should* play in parents’ fulfillment of their duties to their children. With respect to duties owed to potential children—*i.e.*, children existing only prospectively but not actually—the majority of people might instantly agree that “justice requires that the principle be recognized that a child has a legal right to begin life with a sound mind and body” [46]. In other words, the majority of people might affirm that, where a choice of outcomes exist, babies have the right to be born healthy, both mentally and physically; but in whom lies the corresponding duty, and when does such a duty attach?

A potential child possesses not only a legal right to begin life with a sound mind and body, but also an analogous moral right to an “open future”, which suggests that children “possess ‘anticipatory autonomy rights’ that are violated when a child’s opportunities in life are limited” and that “[e]very child that comes into existence has future interests that can be doomed by the child’s circumstances at birth”. Law professor Kristen Rabe Smolensky and philosopher Joel Feinberg argue that, whether in a nonfeasance context—*i.e*., parents forgoing abortion after learning of a fetal disability—or in a misfeasance context—*i.e*., parents using PGD to implant an embryo with a genetic disability—”where parents are fully informed of the likelihood of certain [disabilities], and yet permit a child to be born, they have wronged that child (in a moral sense) even if it cannot be said that the child has been legally harmed” [3].

Such argumentation brings us back to the subjunctive-threshold interpretation of harm, reintroducing the question of what, precisely, “harm” and a satisfactory “reasonable threshold” entail, especially in light of continuously refined RGTs and of the right to disability discussed above. Does new knowledge about genetic risks and interventions impose a new responsibility or obligation on potential parents to use RGTs to ensure that their children evade genetic disability? What leeway will parents maintain in determining which interventions are morally impermissible and which are crucial for protecting their offspring’s right to health and an open future, and how will society moderate such choices among an increasing number of genetic technologies? We will undoubtedly see individual parents deciding, for example, whether selective abortion should be part of their RGT toolkits and how—if at all—they might utilize genetic counseling, with society continuing to reexamine its respective policies (see, e.g., [24]).

However, ultimately, while some individuals and parts of society will continue to resist genetic technologies in favor of more traditional methods of reproduction with minimal or no intervention, others might view the “reasonable threshold” as essentially a moving target, becoming more stringent as RGTs continue being discovered and refined.

Therefore, if we assume the subjunctive-threshold interpretation, we are obliged to determine the “reasonable threshold of harm” and what, specifically, prospective parents and their doctors must do in order to fulfill their duty to prospective children. In other words, we must seek to establish what bodily and mental conditions satisfy the legal and moral rights to health and an “open future”, respectively, and what conditions fall below the reasonable threshold of harm. As we readily see, it is not so simple as asserting that disabilities fall below the reasonable threshold of harm and that all prospective children must be granted minds and bodies devoid of abnormalities. Not only is such a state of health currently impossible to ensure—either through traditional procreation or through RGTs—but it is arguably ethically impermissible. In the following section, this article further explores the notion of the “right to disability”, both for existing people and for prospective children.

## 4. The Right to Disability: A Legal and Ethical Concept

The notion of an ethical right to disability is a strange one. At first glance, one might assume there is no need for such terminology, especially if, as discussed above, we can infer a legal right to life with disability, with corresponding duties mandated by the ADA and other legislation. However, in this age of ever-increasing and ever-refined genetics science and technology that could result in parents’ power to design babies, the ethics of *chosen* disability must be investigated.

As RGTs become more available, whether due to increased affordability or to innovation, potential parents are thrust into an increasingly fraught position of decision-making. Even before gene-editing technology emerged as a real intervention awaiting regulation and application, PGD offered itself—to those who could afford it and were willing to undergo the invasive procedure of *in vitro* fertilization (IVF) [48]—as a way to ensure that only embryos without an identifiable genetic disorder were implanted (see, e.g., [51]). Potential parents’ decision whether to use RGTs such as PGD hinges on such key factors as “whether the embryos would be destroyed, the nature of the disease or trait being avoided or sought, technological control over ‘natural’ reproduction, the value of suffering, disability, and diversity, the importance of having genetically related children, and the type of future people desire[d] or fear[ed]” [49]. Ultimately, PGD has been experienced by potential parents as empowering, even while it subjects many to disappointment and distress [50].

With the emergence of CRISPR/Cas9 and other gene-editing technologies, some of the uncertainties involved with PGD could be evaded, making this type of RGT a clinical “no-brainer”. For example, while PGD can involve the destruction of unimplanted embryos—a concern not explored in this article, but one felt by a good many potential parents—gene-editing technology can circumvent this procedural byproduct: only one embryo need be created—either *in vitro* or through so-called natural reproduction—and then any genetic diseases susceptible to CRISPR/Cas9 technology can be edited out. Furthermore, the low expense and ease of CRISPR/Cas9 grants researchers a “cost-effective and easy-to-use technology to precisely and efficiently target, edit, modify, regulate, and mark genomic loci of a wide array of cells and organisms” [51] and could provide potential parents with an affordable and much more precise way to ensure a genetically healthier baby (as medical observers might view it). However, many of the concerns with PGD enumerated above would remain with CRISPR/Cas9, such as “designer baby” dilemmas, potential devaluing of disability by means of the selecting-out of people with certain conditions, and the hopes and fears harbored by current generations concerning types of future people we could engineer.

These remaining concerns are at risk of being overshadowed by CRISPR’s ease and relative affordability. Will potential parents who can afford to use CRISPR/Cas9, but choose not to, be condemned if they have a baby with a disability? Conversely, would it be ethical for potential parents to effectively eradicate all genetic disabilities susceptible to being edited out? Is there an ethical right to a life with disability when such a life can be avoided by means of gene-editing technology?

These questions cannot be answered before the notion of “disability” is deciphered in a universally satisfactory way—something that might never be able to be accomplished. Since the invention of the cochlear implant, debates have been “simmering over whether certain qualities, including deafness, should actually be considered valuable sources of human diversity instead of disabilities” [20]. In a sense, this diversity debate acknowledges that so-called disabilities can prove to be merely various conditions along a wide spectrum of human existence that prove beneficial to our species, as long as they are not wholly debilitating or make a life not worth living.

Indeed, many individuals who choose so-called disability for themselves or for their children do not perceive the resultant condition as a disability at all. Both deafness and achondroplastic short stature have been viewed as cultural traits, rather than disabilities, by many affected individuals and disability rights activists (see, e.g., [45,46]). Some IVF clinics have reported potential parents requesting the use of PGD to select for either deafness or achondroplasia so that their children may better fit in to the parents’ communities, and some clinics have provided such a service [56], notwithstanding considerable ethical debate as to whether such selection for conditions commonly understood to be disabilities should be permitted.

In essence, “[m]issing from most discussions is a critical assessment of the degree to which genetic research remains reliant upon socially constructed notions of impairment” [57]. Ultimately, a serious tension has emerged between a fear of the “new eugenics” that “can be seen in current attempts to use genetics and biomedical intervention to address complex social problems” [57] and the arguably moral desire to prevent grievous suffering in others due to a genetic condition [58].

We can see that our society defines, protects, and yet fears disability. Although we might agree that we should “extend the concept [of disability] so that it applies broadly across society as a civil right for all—the right to be ill, to be infirm, to be impaired without suffering discrimination or oppression” [15], taking this concept to the next logical step—*i.e*., that *choosing* disability should be a right for potential parents—might not sit well. However, just as certain elected physical alterations—such as transgenderism, transhumanism, and pharmaceutical enhancement via so-called smart drugs—have become normalized to varying degrees, so, too, might the election of disability proliferate as an increasingly common—and increasingly normalized—option for those using genetic engineering.

## 5. Conclusions

Following a three-day International Summit on Human Gene Editing, scientists have called for a moratorium on germline editing. However, they have left the door open to such use of CRISPR/Cas9 after “the risks could be better assessed and until there was ‘broad societal consensus about the appropriateness’ of any proposed change” [59]. As risks are studied and the technology refined, it seems undeniable that there will be a place for genetic intervention, including the use of CRISPR/Cas9 on germ cells, in a therapeutic context. Eradicating genetically identifiable diseases whose cures are currently being sought—such as Huntington’s disease, cystic fibrosis, and sickle cell disease (to name just three)—surely strikes most observers as likely to help, more than hurt, those afflicted. Following the further study called for by the Summit group, any remaining risks of the technology would arguably be deemed acceptable, given the promise of a life free of the respective diseases.

In the meantime, further debate and exploration of the ethical issues are needed, especially with respect to the potential use of CRISPR/Cas9 to ensure certain disabilities, such as deafness, in prospective children so that they can better integrate themselves into a particular community. What would be the broad societal implications of chosen disability?

As noted, the ADA protects the legal rights of individuals with disabilities without regard to etiology; to put the point more expansively, the ADA endows all members of society with the right to invoke protections and duties should a disability arise from any cause. Thus, this article has interpreted the ADA (and the UNCRPD, where ratified) as establishing a right to disability, rooted in law and extending into the ethical domain. The right to disability consists of the right to *incur* disability along with the right to *invoke all protections* for those with disabilities secured by the ADA. But intrinsic to this concept is the extension of the right to *engineer* disability through genetic decision-making—whether through use of PGD, by means of an accidental side effect of CRISPR, or through an application of PGD or CRISPR in quest of a disability (as already seen with PGD). That is to say, one has the right to experience disability caused by either a natural or a technological genetic abnormality, just as one has the more universally agreed-upon right to health.

Resultant questions must include: Which conditions qualify as “disabilities” protected by the ADA and UNCRPD, and which should be deemed non-disabling or cultural traits? Who gets to decide? If a so-called disability is chosen by someone who perceives the condition as a non-disabling trait, should that person still be protected by the ADA/UNCRPD on the basis of how the larger society views the trait? This question bears within it a paradox: could the exercise of the *right to disability* become self-canceling? Or may society, in its effort to maintain protections, impose the status of “disabled” on someone who refutes that label? In so doing, would society be enshrining a “reasonable threshold” whereby the ostensibly disabled person can claim to have been harmed by failure to eliminate the disability?

The notion of harm—and the parental duty not to harm—further illuminates the paradoxical vein running through the ADA and UNCRPD. In seeking to eliminate societal marginalization of people with disabilities, these laws explicitly affirm society’s duty *and capacity* to fully integrate such people; thus, the “harm” that might have attended the engineering of impairments should theoretically be neutralized by appropriate accommodations. Let us look more particularly at the UNCRPD’s commitment to neutralizing the fundamental social problem of stigmatization, as detailed in Article 8 (Awareness Raising), which stipulates the need to “combat stereotypes” and “promote positive perceptions” of people with disabilities. [16] The National Council on Disability (NCD) has identified gaps between the stipulations of the UNCRPD and the rights guaranteed by US federal law, noting for example that “[t]he Individuals with Disabilities in Education Act (IDEA)…does not affirmatively mandate the breakdown of social stigma relating to children with disabilities” [18]. By way of remedy, the NCD suggests, “Congress could utilize its spending powers to encourage larger attitudinal changes by providing subsidies or tax incentives to media outlets, or grants to educational facilities, that worked towards breaking down historical stereotypes relating to disability” [18]. Once we agree with the UNCRPD that stigma is to be combatted, and further agree with the NCD that we possess tools for “breaking down” stereotypes and social stigma, we must reexamine the notion that it is the *parents* who harm a child by choosing traits that an unreconstructed society deems undesirable. We might even go so far as to argue that such parents are using the ultimate tool for combatting social stigma; in fact, to speak of their having “harmed” their child is to reinvigorate stigma against the traits they have autonomously chosen. Ultimately, if we artificially engineer social diversity by allowing CRISPR to be used in the selection of so-called disabling traits, we will be demonstrating that we are not seeking to eliminate the so-called strangeness or otherness of disability.

A reader will perhaps look askance at this inversion of established understandings. I am not, in fact, intending either serious or satirical advocacy of a scenario in which parents might receive societal encouragement to “biodiversify” their offspring in celebration of social diversification. What I am suggesting is that in debating guidelines for application of CRISPR, we must consider the consumers who will ask to put the technology to idiosyncratic use, and who, in doing so, may expose twists of unwitting paradox, verging on self-contradiction, within disability advocacy itself.

If the US were to strengthen or even look beyond the ADA and related disability rights legislation and instill protections against social stigma, the result would be both a legal and social landscape akin to that envisioned by the UNCRPD. By extension, the use of CRISPR to engineer so-called disabling traits would need to be viewed in much the same light—whatever that may turn out to be—as would the use of CRISPR to engineer the eradication of genetic disability or even so-called enhancement. This article has not attempted to define how such a right to disability will or should be understood in relation to reproductive decision-making; rather, it has offered the concept as a lens through which to focus the ongoing CRISPR discussion among scientists, bioethicists, and the public. Throughout this paper the terms “disability”, “impairment”, and “harm” have frequently appeared without quotation marks around them, as if they possessed a commonsense meaning. In every such case, my pragmatic suspension of quotation marks was accompanied by an acute awareness that the confluence of social idealism and biotechnological opportunity demands a careful appraisal of these terms.

Plunged into the ethical vortex of CRISPR, we may be grateful that someone has called a time-out, but we realize that the interlude will be brief. However long the moratorium on CRISPR application, we must vigorously continue the discussion of how best to regulate this technology’s use in addressing what we perceive as disability. Not only must we determine whether and how potential parents use it to eradicate genetic disease, but we must also articulate how CRISPR should interact with the right to disability. Once CRISPR is available and regulated, must potential parents use it to eradicate genetic disease? Can potential parents decide not to use it and permit genetic disease to happen naturally? Can potential parents be permitted to use it to impose disability on their prospective children? Are all putative disabilities equal in the eyes of society, or may we collectively design our population’s range of disability? Ultimately, do we ensure a more egalitarian society by affirming choice for all individuals or by defining, and delimiting, the benefits and harms that may be imposed on future citizens?
